# A Residual Analysis-Based Improved Particle Filter in Mobile Localization for Wireless Sensor Networks

**DOI:** 10.3390/s18092945

**Published:** 2018-09-04

**Authors:** Long Cheng, Liang Feng, Yan Wang

**Affiliations:** Department of Computer and Communication Engineering, Northeastern University, Qinhuangdao 066004, China; zxam1099@yeah.net (L.F.); wangyan_jgxy@neuq.edu.cn (Y.W.)

**Keywords:** wireless sensor network, non-line of sight error, mobile localization, particle filter, residual analysis

## Abstract

Wireless sensor networks (WSNs) have become a popular research subject in recent years. With the data collected by sensors, the information of a monitored area can be easily obtained. As a main contribution of WSN localization is widely applied in many fields. However, when the propagation of signals is obstructed there will be some severe errors which are called Non-Line-of-Sight (NLOS) errors. To overcome this difficulty, we present a residual analysis-based improved particle filter (RAPF) algorithm. Because the particle filter (PF) is a powerful localization algorithm, the proposed algorithm adopts PF as its main body. The idea of residual analysis is also used in the proposed algorithm for its reliability. To test the performance of the proposed algorithm, a simulation is conducted under several conditions. The simulation results show the superiority of the proposed algorithm compared with the Kalman Filter (KF) and PF. In addition, an experiment is designed to verify the effectiveness of the proposed algorithm in an indoors environment. The localization result of the experiment also confirms the fact that the proposed algorithm can achieve a lower localization error compared with KF and PF.

## 1. Introduction

Wireless Sensor Networks (WSN), which can be used in many fields such as urban management, environmental monitoring, disaster-relief, remote control of dangerous places, are a new multi-disciplinary overlapping research area. Compared with the Internet, which provides people with a fast and convenient way to communicate with each other, WSNs combine the logical information world with the real physical world, changing the way human beings and the world interact. A WSN is a comprehensive intelligent information processing platform which integrates sensor technology, microelectromechanical technology, advanced network and wireless communication technology. It has broad application prospects. Its development and application will have a far-reaching influence on all fields of human life. In brief, a wireless sensor network is a computation and communication network of sensors. These sensors are capable of computing and communicating with each other in a wireless way. The data collected by sensors is gathered together so that the information of a scene monitored by the network can be known easily.

Localization is widely applied as a key WSN technology. Due to the flexibility of WSNs, it is a powerful supplement of to the Global Positioning System (GPS), especially when locating indoors targets [1]. In the localization problem context, there are two types of nodes in WSNs: beacon nodes and unknown nodes. The coordinates of a beacon node are known a priori, while the coordinates of unknown nodes which are fixed on personnel or valuable equipment are unknown. There are several ways of localization through WSNs: time of arrival (TOA), time difference of arrival (TDOA), angle of arrival (AOA) and received signal strength (RSS). The TOA scheme obtains the distance between the unknown node and the beacon node through the propagation time, and once there are three or more beacon nodes the unknown node can be located. The TDOA scheme obtains the distance difference between the unknown node to any two different beacon nodes through the time difference of signal propagation between two beacon nodes and with three or more beacon nodes the unknown node can be located. The AOA scheme uses the angle of arrival. It is a high precision schem but it needs the extra hardware such as an antenna array. RSS is a low cost and low power consumption solution, but the localization accuracy is also lower compared with the other methods.

The above scheme can achieve high localization accuracy in an ideal environment. However, in practical scenarios there must be some errors. These errors can be simply summarized into two types. One of them is the measurement errors. These errors result from system noise or measurement noise which are also exist in many other subjects. Another error is the Non-Line-of-Sight (NLOS) error. Compared with the first type of error, NLOS error decreases the localization performance more severely. As the major challenge of localization, NLOS errors occur when the propagation of the signal is obstructed by some unknown obstacle. The NLOS error obeys different distribution functions for different scenes. In order to eliminate the negative effect of NOLS error, many algorithms are proposed. In general, these algorithms can be divided into two categories. One is detecting and selecting the measurements without NLOS error. The other is weighting the data without figuring out its propagation status.

In this paper, we adopt both ideas in the proposed algorithm. Firstly, we generate some particles around the target. By conducting a two-time residual analysis-based selection, some reasonable particles are picked out to locate the target. Then, these selected particles are weighted by their residuals to launch the localization. Thanks to the selection, the negative impact of NOLS error is decreased and a reasonable position of the target is figured out through the particles. Besides, the system error and the measurement error are also reduced by using the weighting algorithm in localization. The main contribution of this paper is listed as follows:

(1) The residual analysis is used several times to ensure the effectiveness of the localization results.
(2) The beacon nodes work together so that the particle filter process is simplified and the negative effect of NLOS errors can be decreased. The computational complexity of the proposed method is lower than that of the PF algorithm.
(3) The proposed algorithm doesn’t make any assumption on the distribution of the NLOS error.

This paper is structured as follows: in Section 2, related works in localization technology are analyzed. The signal model and some background knowledge are illustrated in Section 3 and Section 4 explains the proposed algorithm in detail. The simulations and experimental results of the proposed method are shown in Section 5. Conclusions are drawn in Section 6. Some key notations are explained in Table 1.

## 2. Related Works

As localization through WSNs is playing an increasingly important role in modern society, many methods have been studied by researchers. These papers can be divided basically into two categories. One of those identifies the propagation of the signal. Shi utilized the channel sight information to improve the localization accuracy in [2]. A relocalization algorithm is used in this method to review the identified channel sight conditions so that the error of position estimation can be reduced. Zhang focused on identifying NLOS components by characterizing the acoustic channels in [3]. By analyzing indoor acoustic propagations, the changes of acoustic channel from the line-of-sight (LOS) condition to the NLOS condition are characterized. Then, an efficient approach to estimate relative channel gain and delay based on the cross-correlation method is proposed. Finally a support vector machine (SVM) classifier with a radial-based function (RBF) kernel is used to realize NLOS identification. Yan adopted a three-step localization approach in [4]. A Bayesian sequential test is designed to figure the measurement data with NLOS effect. A modified Kalman filter (MKF) is used to smooth the measurement range and mitigate the NLOS effect. After adjusting the measurement noise covariance and prediction covariance by an established measurement equation, a residual weighting algorithm is used to finish the final estimated target position. Ma presented an indoor localization method based on angle of arrival and phase difference of arrival (PDOA) in [5]. An antenna array is used to distinguish multipath signals and two strongest paths are chosen to perform localization. Using the angles and distances, virtual stations are established to convert NLOS paths into LOS paths. Finally, after possible positions of the tag are calculated, the weighted least squares combined with residual weighted algorithm are used to calculate real position of the tag. Choi proposed a recurrent neural network (RNN) model in [6]. The RNN model takes a series of channel state data to identify the corresponding channel condition. Numerous measurement data from an indoor office environment are trained. Performances of existing schemes that use handcrafted features are also compared. The proposed method yields high accuracy. Pak proposed a new NLOS identification algorithm based on a distributed filter in [7]. The proposed algorithm uses distributed filtering and data association techniques to identify abnormal measurements due to NLOS so that negative effects can be prevented. Besides, the hybrid particle finite impulse response filter (HPFF) was adopted. The proposed algorithm can mitigate NLOS effects by identifying NLOS situations and self-recovering from failures. Gazzah combined received signal strength and angle of arrival (RSS/AOA) measurements to solve the problem of LOS/NLOS identification in [8]. By proposing a hybrid hypothesis test (HHT), the most probable two base stations (BSs) to be LOS can be identified. If two LOS BSs are identified, localization proceeds directly. Otherwise, a weighting approach will be applied. Pandey proposed an algorithm using visible light to identify NLOS condition in [9]. Using the time difference of arrival in a maximum likelihood framework, the reflection points are first localized. Then, by applying novel geometric methods from range and reflection angles the location of the sensor nodes is then estimated. Shi proposed to use Maximum Likelihood Estimator (MLE) for localization in [10]. The algorithm utilizes all the available measurements and explicitly takes the probabilities of occurrences of LOS and NLOS propagations into account. Yang proposed a localization algorithm based on Import Vector Machine (IVM) and a novel NLOS identification algorithm with feature selection strategy in [11]. The classification accuracy is ameliorated by a feature selection strategy. By employing the probability outputs of IVM, the localization algorithm yields higher positioning accuracy. Momtaz proposed a novel algorithm to identify and eliminate the NLOS error based on subspace method in [12]. Simulations showed that the algorithm is faster and more accurate compared with conventional methods, especially in a large-scale environment.

Some other algorithms conduct localization without identify the propagation status. Among these algorithms, Tomic proposed a localization method using RSS and AOA [13]. In this paper, a comprehensive study of the state-of-the-art (SoA) solutions is presented. The localization problem is converted into a generalized trust region sub-problem (GTRS) framework [14]. The computational complexity of this algorithm is linear with the number of reference nodes. Simulation results confirm the capacity of new algorithm of possessing a steady NLOS bias mitigation. Abu-Shaban proposed a two-stage closed-form estimator to localize an MS by three base stations in cellular networks [15]. A distance-dependent bias model is adopted to derive a range estimator as a first step. Then the trilateration is used to find an estimate of the MS position. Ding proposed a new convex optimization model which is built upon the concept of Euclidean distance matrix (EDM) in [16]. The resulting EDM optimization is conducive to algorithm developments. The EDM model outperforms the existing SDP model and several others. Wang proposed a multi-domain features based device-free wireless sensing system in [17]. In order to characterize the block distributions, the time domain and frequency domain measurement matrices are partitioned into basic structure blocks. By considering the spatial relationship between adjacent blocks, coherence histograms are adopted to characterize the distribution of the blocks. Yang proposed a novel NLOS mitigation method based on Sparse Pseudo-input Gaussian Process (SPGP) whose complexity is lower than Gaussian Process (GP) regression in [18]. The approach directly mitigates the bias of both LOS and NLOS conditions. With less training data, SPGP can achieve performance comparable to GP regression. Vilà-Valls proposed a robust Bayesian inference framework to deal with target localization under NLOS conditions in [19]. The proposed algorithm takes advantage of the conditionally Gaussian formulation of the skew *t*-distribution. The proposed algorithm is also being able to use computationally light Gaussian filtering and smoothing methods. Numerical results show the performance improvement of the proposed algorithm. Wang developed a novel robust optimization approach to source localization in [20]. The approach uses time-difference-of-arrival (TDOA) measurements which are collected under non-line-of-sight (NLOS) environment. Instead of obtaining the distribution or statistics of the NLOS errors, the approach only assumes that the NLOS errors have bounded supports. Then, formulating the TDOA-based source localization issue as a robust least squares (RLS) problem. Besides, in this paper, two efficiently implementable convex relaxation-based approximation methods are proposed since the RLS problem is non-convex. Park utilized a weighted least squares (WLS) method to deal with line-of-sight (LOS)/non-line-of-sight (NLOS) mixture source localization problem in [21]. Error covariance matrix is used for the sample mean and median to minimize the weighted squared error (WSE) loss function. Via simulation compared with the mean square error (MSE) performance, the superiority of the proposed methods is verified. Li used a two-state Markov chain to represent the switching behavior of the LOS/NLOS time-of-arrival (TOA) measurements in [22]. The algorithm cast the cooperative localization problem into a state estimation for a class of jump Markov nonlinear system. By applying the interacting multiple model (IMM) method and the extended Kalman filter (EKF) technique, a multi-sensor multi-model filter has been developed. Gaber proposed properly weighted least square (WLS) and hybrid WLS estimators which are used to mitigate the effect of undetected direct path channel profiles in [23]. The paper also presented an adaptive method to select the reference base station (BS) and extract certain weights so that the observations based on the estimated channel profiles can be scaled. A semidefinite programming (SDP) relaxation based method is proposed by Biswas in [24]. The algorithm requires very few anchor nodes to estimate the position of the unknown node. The algorithm can also achieve minimal estimation errors even when the anchor nodes are randomly deployed. He proposed an indoor localization method combining the strengths of trilateration and fingerprinting in [25]. An optimization formulation following the spirit of the trilateration helps to find the target location. By some experiments in different place, the effectiveness of the algorithm is confirmed. Based on received signal strength (RSS) and angle of arrival (AoA) measurements [26], Tomic proposed a Bayesian methodology for target tracking in [27]. The proposed algorithm performs better than the algorithms which uses only information from observations. In another paper [28], Tomic linearizes the measurement models and incorporates the prior knowledge obtained from target state transition model. Simulation results confirm the efficacy of the proposed algorithms.

When the propagation is known a priori, Wang improved the performance of the existing robust weighted least squares (RWLS) method in [26]. In his paper, a procedure of correcting the incorrect path status which makes the RWLS method robust against the path status identification errors is proposed so that the algorithm performance will not degraded when the path status is incorrectly identified. In his other paper [27], Wang impose a weight to the term with respect to the NLOS measurements so that more accurate LOS measurements are fully utilized. Then, a second-order cone relaxation technique is adopted to relax the problem as a tractable second-order cone program.

## 3. Problem Statement

### 3.1. Signal Model

**Figure 1:**
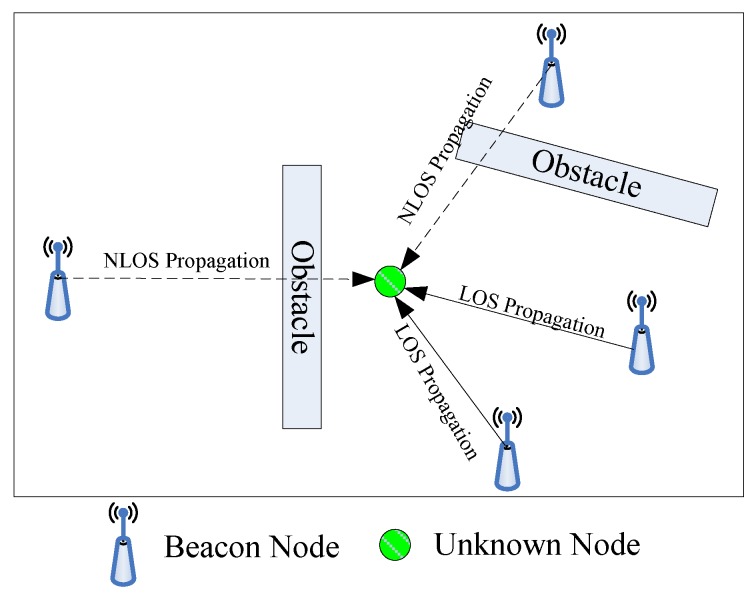
The localization result of the proposed algorithm.

As shown in Figure 1, we assume there are *N* beacon nodes deployed randomly in a certain scenario in which an unknown node is to be located. The coordinate of each beacon node is known a priori and can be represented as $\left[x_{i},y_{i}\right]$ (i=1,2,...,N). The coordinates of the unknown node are unknown and represented as [x,y]. When the direct propagation path between the beacon node and unknown node is blocked, the propagation state is NLOS propagation.

Thus, the true distance between *i-*th beacon node $\left[x_{i},y_{i}\right]$(i=1,2,...,N) and the unknown node at time *k* can be represented by the following expression:

(1) $$d_{i}^{k}=\sqrt{{\left(x_{i}-x\right)}^{2}+{\left(y_{i}-y\right)}^{2}}$$

The above equation describes the distance between *i-*th beacon node and the unknown node in an ideal scenario. However, there are some noises in the practical scenario, including system noise and measurement noise. In LOS state, the measuring distance between *i-*th beacon node and the unknown node is given by:

(2) $${\hat{d}}_{i}^{k}=\sqrt{{\left(x_{i}-x\right)}^{2}+{\left(y_{i}-y\right)}^{2}}+\varepsilon$$

where, ε is the measurement error which follows a zero-mean Gaussian distribution with standard deviation $\sigma_{i}^{2}$.

In some complex environments, the direct path between the beacon node and unknown node may be blocked by an obstacle. The signal propagation ways are reflection, diffraction or refraction. The actual propagation distance will increase. Therefore, the measuring distance between *i*-th beacon node and the unknown node in NLOS state as [29,30]:

(3) $${\hat{d}}_{i}^{k}=\sqrt{{\left(x_{i}-x\right)}^{2}+{\left(y_{i}-y\right)}^{2}}+\varepsilon +\varepsilon_{NLOS}$$

where, $\varepsilon_{NLOS}$ is the NLOS error, it obeys different distribution in different scenario including Gaussian, uniform or exponential distribution.

When NLOS error obeys Gaussian distribution $\varepsilon_{NLOS}\sim N\left(\mu_{NLOS},\sigma_{NLOS}^{2}\right)$, it can be described as:

(4) $$f\left(\varepsilon_{NLOS}\right)=\frac{1}{\sqrt{2\pi \sigma_{NLOS}^{2}}}\exp\left(-\frac{{\left(\varepsilon_{NLOS}-\mu_{NLOS}\right)}^{2}}{2\sigma_{NLOS}^{2}}\right)$$

When the NLOS error obeys a uniform distribution $\varepsilon_{NLOS}\sim U\left(u_{\min},u_{\max}\right)$, it can be described as:

(5) $$f\left(\varepsilon_{NLOS}\right)=\{\begin{array}{cc} \frac{1}{u_{\max}-u_{\min}} & ,for\;u_{\min}\leq \varepsilon_{NLOS}\leq u_{\max}\\ 0 & ,\;else \end{array}$$

When the NLOS error obeys an exponential distribution $\varepsilon_{NLOS}\sim E\left(\lambda \right)$, it can be described as:

(6) $$f\left(\varepsilon_{NLOS}\right)=\{\begin{array}{cc} \lambda^{-1}e^{-\varepsilon_{NLOS}/\lambda } & ,\;\varepsilon_{NLOS}\geq 0\\ 0 & ,\;\varepsilon_{NLOS}<0 \end{array}$$

**Figure 2:**
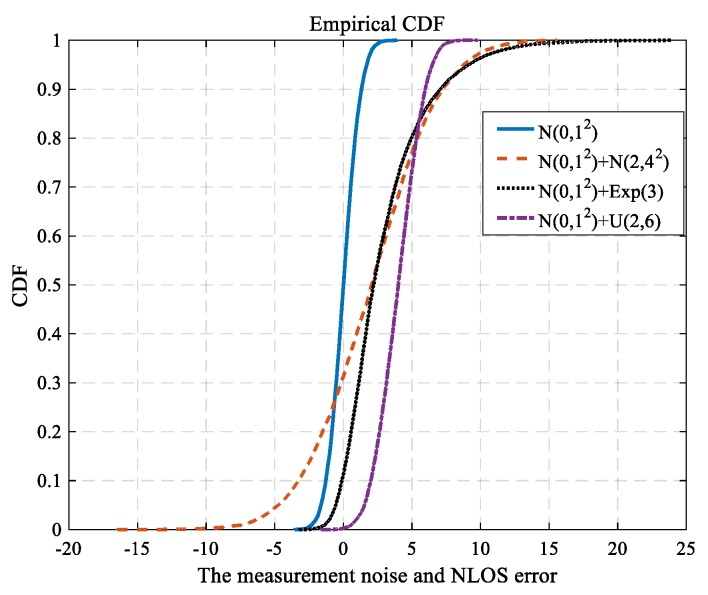
The CDF for measurement noise and NLOS error.

Figure 2 shows the cumulative distribution function (CDF) of measurement noise and NLOS error. The measurement noise ε obeys a Gaussian distribution, i.e., ε~*N*(0, 1^2^). The NLOS error is uniform distribution, Gaussian distribution or experimental distribution, i.e., $\varepsilon_{NLOS}$~*U*(2, 6), $\varepsilon_{NLOS}$~*N*(2, 4^2^), $\varepsilon_{NLOS}$~*E*(3).

### 3.2. A Brief Introduction to PF

The particle filter (PF) was developed in the late 1990s. It is another form of implementation of recursive Bayesian filtering [31]. The idea of PF is to use some random sample points to describe the probability distribution of the target. These sample points are called particles. Then, by adjusting the weight of each particle and the location of the sample points, the estimated value of the unknown node can be obtained through weighted value of the sample. It can be applied to any non-linear non- Gauss stochastic system in principle.

Based on Bayesian estimation theory and the Monte Carlo method, PF is reckoned as the most representative non-linear filtering implementation method. PF uses a bunch of particles to fit the target. Those particles which fit the target closely get bigger weight, and then get copied. After several rounds of filtering, particles can achieve a good fit to the target. PF doesn’t make any assumption about the probability density distribution of the NLOS error. When it comes to large measurement noise, PF can achieve better results than the Kalman Filter and other filtering algorithms. There are three major steps in particle filter. Firstly, PF generates a set of particles around the target as a prediction. By calculating the distance difference between the target to the node and particle to the node, the weight of the particles is given. Secondly, those particles with small weight are discarded. At the same time, those particles with big weight are copied. Then, the reserved and the copied particles undergo a weight normalization. After the normalization, the particles are used to locate the target. Finally, all particles adopt a random movement in a small range so that the particles will not gather together. These particles are prepared for the next localization.

## 4. Proposed Method

### 4.1. General Concept

In this paper, we assume that there are *N* beacon nodes and *P* particles to locate a mobile unknown node. The coordinate of the *p-*th particle can be represented as $\left[X_{p}^{k},Y_{p}^{k}\right]$. As mentioned in Section 3, the measuring distance between the *i-*th beacon node and the unknown node at time *k* is described as ${\hat{d}}_{i}^{k}$. The distance between *i-*th beacon node and *p-*th particle at time *k* can be described as $d_{i,p}^{k}$. And then according to the distance difference, the weight of *p-*th particle to *i-*th beacon node at time *k* is given as $W_{i,p}^{k}$.

**Figure 3:**
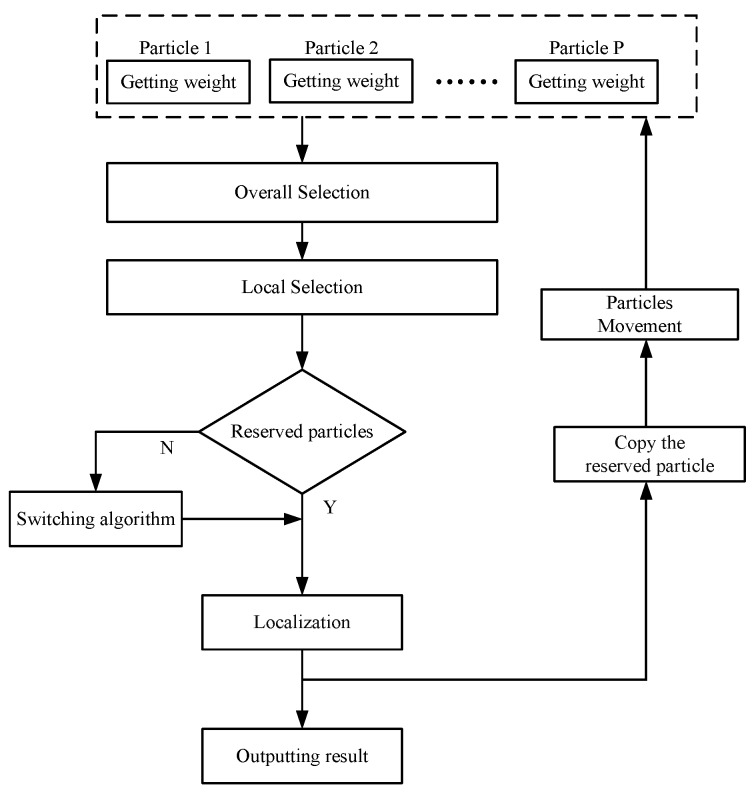
Flow chart of the proposed algorithm.

The flow chart of the proposed algorithm is presented in Figure 3.

### 4.2. Weighting Particles

The first step of the algorithm is to weight each particle. The weight of particles is directly concerned with the localization result. The proposed algorithm weights particles by residual analysis rather than the normal PF which weights particles from a Gaussian function. The proposed algorithm’s weighting process can be divided into three steps:

Firstly, calculate the distance difference from the *p-*th particle to the *i-*th beacon node and the unknown node to *i-*th beacon node at time *k*. The distance difference between them is defined as the residual of the *p-*th particle to the *i-*th beacon node at time *k*, which can be represented as $res_{i,p}^{k}$. It can be given by:

(7) $$res_{i,p}^{k}=\left|{\hat{d}}_{i,p}^{k}-{\hat{d}}_{i}^{k}\right|$$

Secondly, we calculate the residual of the *p-*th particle to every beacon node and sum them. This is defined as the residual of the *p-*th particle at time *k*, which can be represented as$\;res_{p}^{k}$. It can be given by:

(8) $$res_{p}^{k}=\sum_{i=1}^{N}\left|{\hat{d}}_{i,p}^{k}-{\hat{d}}_{i}^{k}\right|$$

Finally, obtain the reciprocal of the residual of the *p-*th particle at time *k*, and the weight of *p-*th particle at time *k* is acquired. It can be given by:

(9) $$W_{p}^{k}=1/res_{p}^{k}$$

The pseudo code Algorithm 1 can be summarized as follows:

**Algorithm 1.** Weighting particles.$input\;\text{:}\;{\hat{d}}_{i,j}^{k},{\hat{d}}_{i}^{k}$ $output\;\text{:}\;W_{j}^{k}$ *for j* = 1:*P* $res_{j}^{k}=0$ *for i* = 1:*N*     $res_{i,j}^{k}=abs\left({\hat{d}}_{i,j}^{k}-\;{\hat{d}}_{i}^{k}\right)$     $res_{j}^{k}=res_{i,j}^{k}+res_{j}^{k}$    *end for*    *for j* = 1:*P*     $W_{j}^{k}=1/res_{j}^{k}$    *end for* *end for*

### 4.3. Overall Selection

Once the weights of all particles are given, the algorithm launches its first selection. Since the weight represents the likelihood that a particle fits the unknown node, the reasonable particle should get a bigger weight. Therefore, the first selection picks up some bigger weight particles, and the threshold is defined as the average weight of these particles. Assuming there are in total *S* numbers of the particles which are selected from the first selection, the pseudo code Algorithm 2 can be summarized as follows:

**Algorithm 2.** Overall Selection. $input\text{:}\;particles\;and\;their\;weight\;W_{i}^{k}\left(i=1,2,...,P\right)$ $output\text{:}\;particles\;and\;their\;weight\;W_{i}^{k}\left(i=1,2,...,S\right)$ *for j* = 1:*P*
 $if\;\left(W_{j}^{k}\geq \sum_{i=1}^{P}W_{i}^{k}/P\;\right)$   *particle reserved;*   *else* *particle discard;* *end for*


The selected particles are considered reasonable because the weight is bigger. However, once several beacon nodes judge the particle improperly, the particle can also get a bigger weight and pass the first selection. Therefore, the first selection is not a complete judgement. A second selection must be added to confirm these particles. 

### 4.4. Local Selection

After the first selection, some particles of bigger weight are reserved. These particles are reasonable in an overall level. In contrast to the first selection, the second selection is conducted in a local level. Since the step of weighting remains the residuals of the particles, these residuals can be used to estimate the probability of the NLOS error. The residual which is bigger than the average value of these residuals is assumed to be involved with NLOS error. The percentage of these residuals is considered as the probability of the NLOS error, and it can be recorded as λ. Therefore, there should be about N⋅(1−λ) numbers of beacon nodes which are smaller than the average value of these residuals for its LOS propagation. The residual of particles to some certain beacon node can also become large when it is far away from the unknown node. Once it is bigger than the average value of residuals, it is considered as unreasonable. It will be discarded. The second selection as a supplement of the first selection avoids the mistake which can be made in first selection cause a local optimum. The pseudo code Algorithm 3 can be summarized as follows:

**Algorithm 3.** Local Selection. *Input: Reserved particles from Selection I and their weight* *Output: Particles and their weight*
$W_{i}^{k}\left(i=1,2,...,Q\right)$ *m* = 0; *for i* = 1:*N*  *for j* = 1:*P* $if\left(res_{i,j}^{k}\;\geq \sum_{p=1}^{P}res_{p}^{k}/P*N\right)$ *m* = *m* + 1; *end for* *end for* λ=m/P∗N thr=N∗(1−λ) *for j* = 1:*P* *n* = 0; for *i* = 1:*N* $if\left(res_{i,j}^{k}\;\leq \sum_{p=1}^{P}res_{p}^{k}/P*N\right)$    *n* = *n* + 1;  *end for* *if* (*n*>*thr*)  *particle reserved*; *else*  *particle discard*; *end for*

### 4.5. Location Estimation

When the two-time selection is done, the algorithm uses the reserved particles to locate the unknown node. Before the localization starts, the number of reserved particles should be checked. If the propagation state is very bad, most propagation between beacon nodes and unknown node is under NLOS status, the second selection will deny the validity of the selected particles from the first selection. The contradiction between the first selection and the second selection results in no particles being left after the two-time selection. Then, the algorithm conducts localization using the particles selected from the first selection. Otherwise, those particles which pass the two-time selection are reserved to locate the unknown node. Assuming there are *L* particles reserved after the two-time selection, where *L = S* when the contradiction between the first selection and the second selection happens or *L = Q* when there is no contradiction between the first selection and the second selection.

Before the localization starts, the weight of the reserved particles should be normalized. The normalized weight of the *p-*th particles at time *k* is represented as $NW_{p}^{k}$. As for *i-*th beacon node, the distance $dis_{i}^{k}$ between it and the unknown node at time *k* is fit from the weights of the reserved particles and the distance from the reserved particles to the *i-*th beacon node.

The estimated distance $dis_{i}^{k}$ can be given by:

(10) $$dis_{i}^{k}=\sum_{p=1}^{L}NW_{p}^{k}*\;{\hat{d}}_{i,p}^{k}$$

Then maximum likelihood is employed to get the coordinates of the unknown node. The maximum likelihood method can be explained by the following equations. Firstly, based on the coordinate of the unknown node and the beacon nodes the formula which illustrate the distance between the node and the unknown node can be written as follows:

(11) $$\{\begin{array}{c} {\left(x_{1}-x\right)}^{2}+{\left(y_{1}-y\right)}^{2}={\left(dis_{1}\right)}^{2}\\ \vdots \\ {\left(x_{N}-x\right)}^{2}+{\left(y_{N}-y\right)}^{2}={\left(dis_{N}\right)}^{2} \end{array}$$

Then, it can be simplified as follows:

(12) $$\{\begin{array}{c} 2x\left(x_{1}-x_{2}\right)+2y\left(y_{1}-y_{2}\right)={\left(dis_{2}\right)}^{2}-{\left(dis_{1}\right)}^{2}-\left(x_{2}^{2}+y_{2}^{2}\right)+\left(x_{1}^{2}+y_{1}^{2}\right)\\ \vdots \\ 2x\left(x_{1}-x_{N}\right)+2y\left(y_{1}-y_{N}\right)={\left(dis_{N}\right)}^{2}-{\left(dis_{1}\right)}^{2}-\left(x_{N}^{2}+y_{N}^{2}\right)+\left(x_{1}^{2}+y_{1}^{2}\right) \end{array}\;$$

Representing it by a linear equation AX=B, where A and B are defined by:

(13) $$A=2\left[\begin{array}{ccc} \left(x_{1}-x_{2}\right) & & \left(y_{1}-y_{2}\right)\\ \left(x_{1}-x_{3}\right) & & \left(y_{1}-y_{3}\right)\\ \vdots & & \vdots \\ \left(x_{1}-x_{N}\right) & & \left(y_{1}-y_{N}\right) \end{array}\right]\;B=\left[\begin{array}{c} {\left(dis_{2}\right)}^{2}-{\left(dis_{1}\right)}^{2}-\left(x_{2}^{2}+y_{2}^{2}\right)+\left(x_{1}^{2}+y_{1}^{2}\right)\\ {\left(dis_{3}\right)}^{2}-{\left(dis_{1}\right)}^{2}-\left(x_{3}^{2}+y_{3}^{2}\right)+\left(x_{1}^{2}+y_{1}^{2}\right)\\ \vdots \\ {\left(dis_{N}\right)}^{2}-{\left(dis_{1}\right)}^{2}-\left(x_{N}^{2}+y_{N}^{2}\right)+\left(x_{1}^{2}+y_{1}^{2}\right) \end{array}\right]$$

Finally, the estimated coordinate of the unknown node is:

(14) $${\left[x,y\right]}^{T}={\left(A^{T}A\right)}^{-1}A^{T}B$$

### 4.6. Particle Copy and Movement

When the localization is finished, the reserved particles are going to be used in the next localization. Therefore, a copy layer is constructed to decide the particle copy progress. The length of each layer is the value of particle’s weight. Due to the weight normalization, the total length of the constructed layer is 1. As the layer is constructed, a random number whose value obeys uniform distribution from minimum value zero to maximum value one is generated. The random number falls into a certain layer. Then the corresponding particle copies for one time. The copy process continues for *P* times which are the total number of the particles. The scheme ensures the particles with bigger value of weight got more chance to be copied and avoid the situation of local optimum.

The pseudo code Algorithm 4 can be summarized as follows:

**Algorithm 4.** Particle copy. $setting\;Layers\;L=\left(0,NW_{1}^{k},NW_{1}^{k}+NW_{2}^{k},...,\sum_{i=1}^{L-1}NW_{i}^{k},1\right)$ *for m* = 1:*P*
  *Generate rand number r;* *for j* = 1:*L*    $if\left(r<L_{j+1}\right)$
     *copy particle j*;     *break;* *end for* *end for*

When the copy processing is completed, the new particles take a small range movement in which the variance of coordinate of the reserved particles obeys a zero-mean Gaussian distribution with standard deviation $\sigma_{p}^{2}$, avoiding the gathering of these particles. Particles now can be used for the next localization. The movement of particles can be given by:

(15) $$\left(X_{p}^{k+1},Y_{p}^{k+1}\right)=\left(X_{p}^{k},Y_{p}^{k}\right)+N\left(0,\sigma_{p}^{2}\right)$$

## 5. Simulation and Experiment Results

### 5.1. Simulation Results

In this section, we evaluate the performance of the proposed residual analysis-based improved particle filter (RAPF) algorithm. We compared the proposed method with the Kalman filter (denoted as KF) and particle filter (denoted as PF). The simulation is performed using MATLAB. The position of beacon nodes for each Monte Carlo run is uniformly deployed in the 100 m × 100 m square space. The propagation condition between the unknown node and beacon node is generated randomly with probability α. The simulation results are obtained over 1000 Monte Carlo runs. We consider the Root Mean Square Error (RMSE) as the performance metric, it can be given by:

(16) $$\mathrm{RMSE}=\sqrt{\frac{1}{K\cdot t_{n}}\sum_{i=1}^{t_{n}}\sum_{k=1}^{K}\left({\left(x\left(k\right)-{\hat{x}}_{i}\left(k\right)\right)}^{2}+{\left(y\left(k\right)-{\hat{y}}_{i}\left(k\right)\right)}^{2}\right)}$$

where $t_{n}$ = 1000, *K* = 100, [x(k),y(k)] is the true position of the target at time *k*, and $\left[{\hat{x}}_{i}\left(k\right),{\hat{y}}_{i}\left(k\right)\right]$ is the estimated position for the *i-*th run at time *k*.

#### 5.1.1. The NLOS Errors Obey a Gaussian Distribution

The default parameter values in the simulation are shown in Table 2.

**Figure 4:**
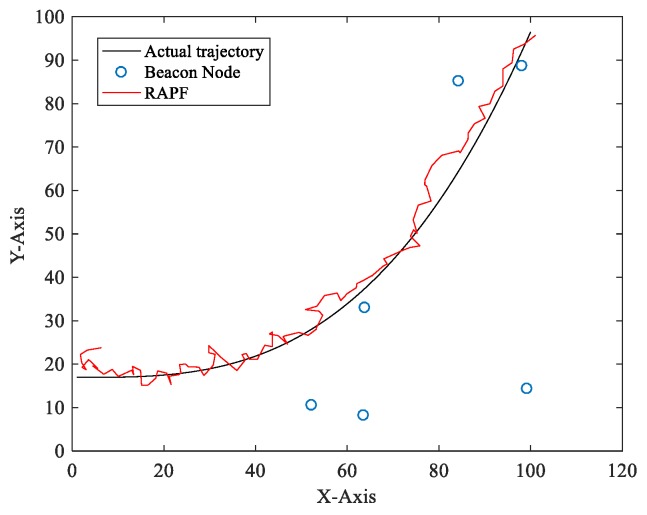
The localization result of RAPF.

Figure 4 shows a one-time localization result of RAPF. As we can see from the picture, the proposed algorithm achieves a reasonable result even when the beacon nodes are deployed randomly.

**Figure 5:**
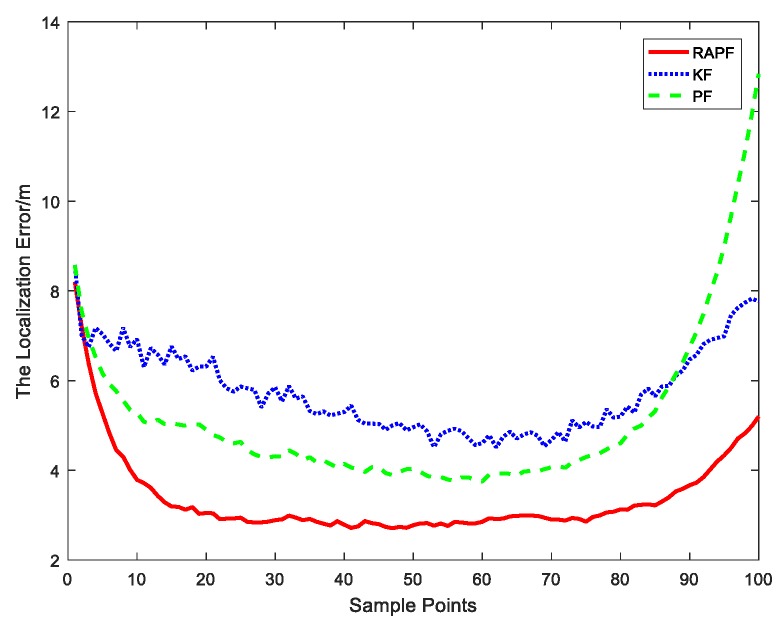
The localization error at each sample points.

Figure 5 reveals the localization error of each sample point. Compared with the KF and PF algorithms, the proposed algorithm displays a lower localization error.

**Figure 6:**
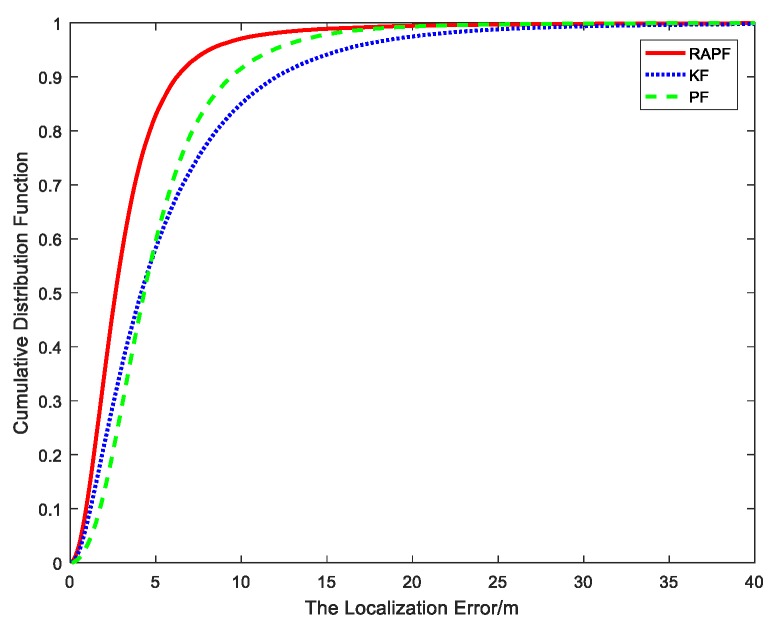
The cumulative distribution error of the localization error.

Figure 6 shows the cumulative distribution function of the localization error. It can be seen that ninety percent of the errors of the RAPF are less than 6.2 m. In contrast, the errors of KF and PF are 12 m and 9.3 m, respectively.

**Figure 7:**
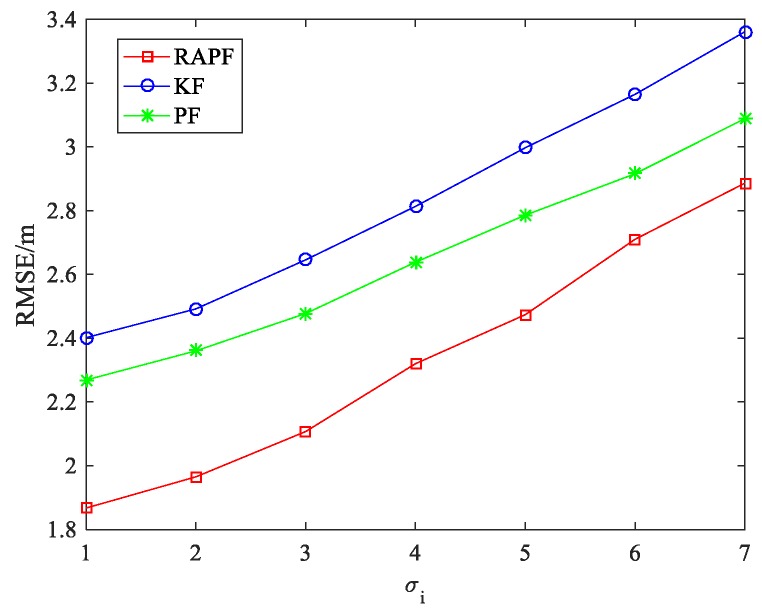
The RMSE versus the standard deviation of measurement noise $\sigma_{i}$.

The standard deviation of measurement noise $\sigma_{i}$ is also considered. As we can see from Figure 7, the RMSE increases when the $\sigma_{i}$ changes from 1 to 7. The performance of the RAPF is better than that of the other two algorithms. Compared with the KF and PF algorithms, the localization accuracy of RAPF is improved about 17.841% and 11.897%, respectively.

**Figure 8:**
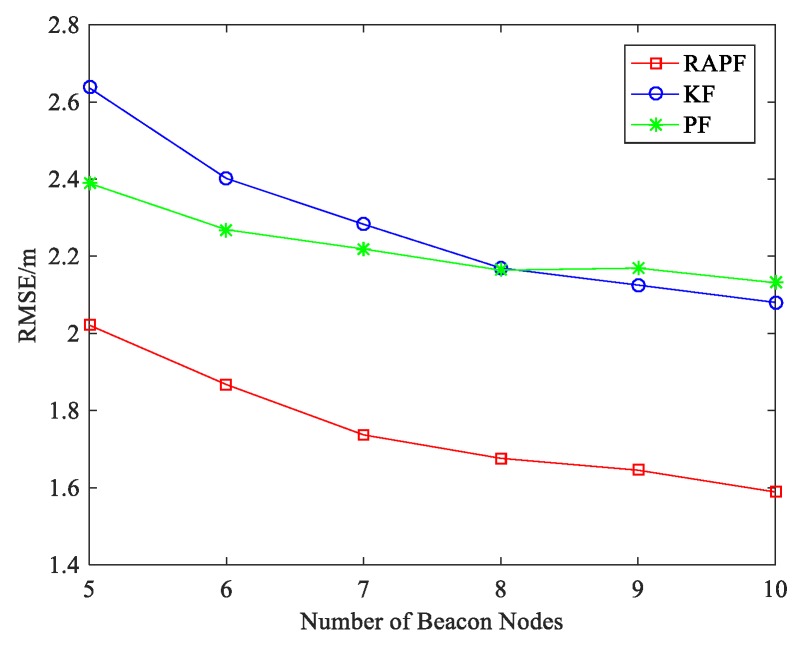
The RMSE versus the number of beacon nodes.

Figure 8 shows the impact of number of beacon nodes on localization error. Compared with KF and PF, the RAPF algorithm has better localization result as the number of beacon nodes increases. As the number of beacon nodes increases, more beacon node re under LOS condition in which KF will get a better localization result than PF. When the number of beacon nodes is 5, the localization accuracy of RAPF improves by 23.336% and 15.393% compared with KF and PF.

**Figure 9:**
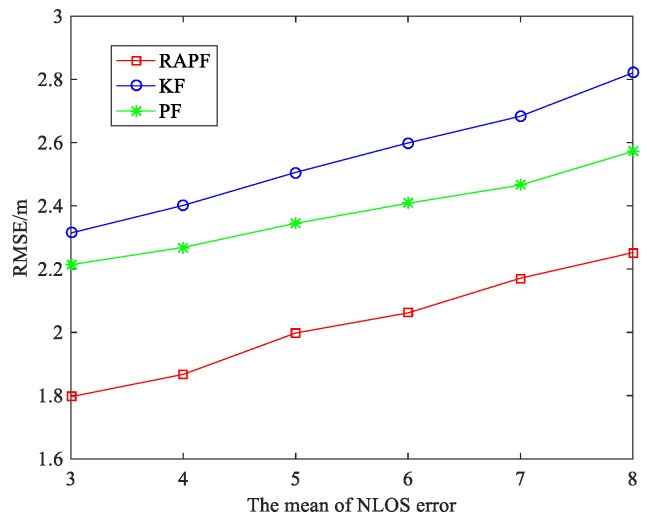
The RMSE versus the mean of NLOS error.

**Figure 10:**
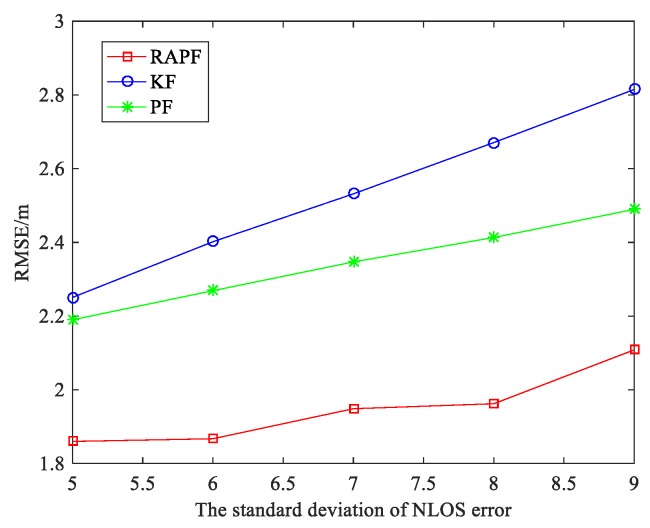
The RMSE versus the standard deviation of NLOS error.

To further evaluate the performance of the RAPF algorithm, we compare the RMSE as the mean and standard deviation of NLOS error in Figure 9 and Figure 10. It can be seen that the localization errors of all three algorithms increase with the NLOS error increases. As shown in Figure 9, the average RMSE of RAPF algorithm is 2.024 m, however the average RMSE of KF and PF algorithm are 2.554 and 2.379 m. In Figure 10, the RMSE of RAPF increases slowly compared with KF. When the standard deviation of NLOS error is larger ($\sigma_{NLOS}=9$), the localization accuracy of RAPF improves 25.093% and 15.307% compared with KF and PF algorithms.

**Figure 11:**
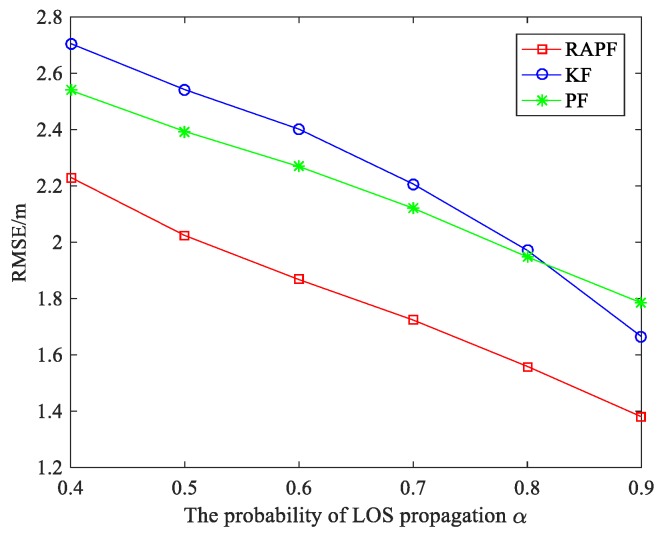
The RMSE versus the probability of LOS propagation α.

Figure 11 shows the impact of the probability of LOS propagation α on the localization error. It can be observed that the α values have a significant impact on localization error. 

The localization error decreases with the α increases. This is because the larger α, the less the NLOS interference is. The localization accuracy of RAPF improves by 20.067% and 17.392% compared with KF and PF.

#### 5.1.2. The NLOS Errors Obey a Uniform Distribution

The default parameter values in the simulation are shown in Table 3.

**Figure 12:**
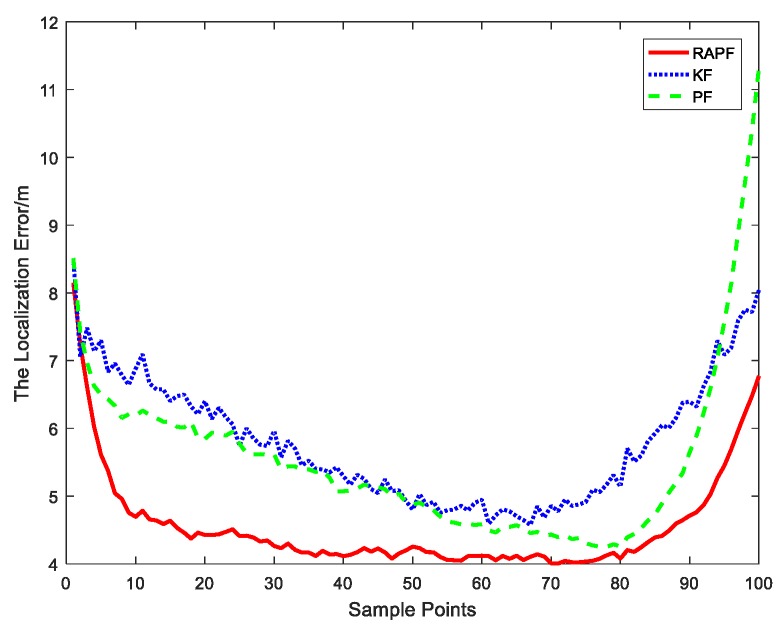
The localization error at each sample points.

**Figure 13:**
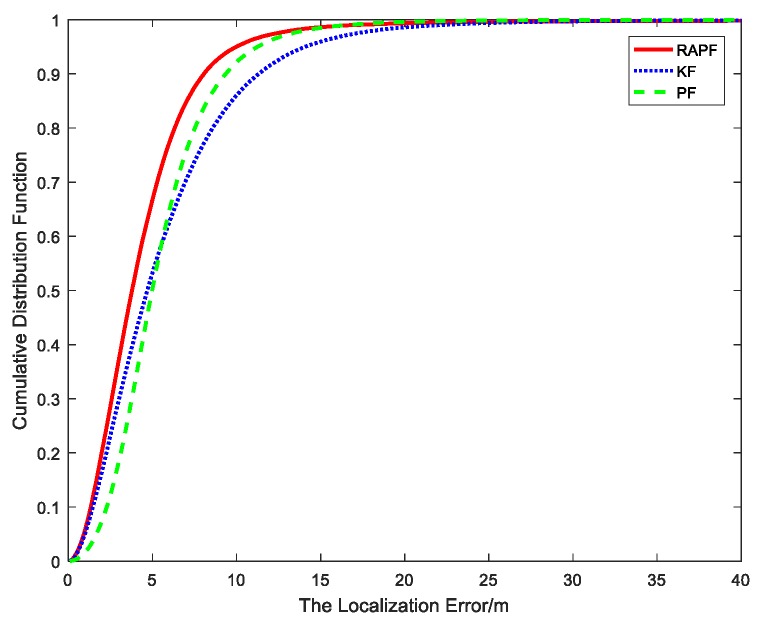
The CDF versus the localization error.

Figure 12 and Figure 13 show the localization error when the NLOS error obeys a uniform distribution, i.e., $\varepsilon_{NLOS}\sim U\left(2,12\right)$. Figure 12 shows the relationship between the localization error and sample points. It can be observed that the proposed method possesses the highest localization accuracy for the most sample points. Figure 13 shows the cumulative distribution function of the localization error. Ninety percent of the errors in RAPF are less than 8 m. In contrast, the Kalman filter and particle filter errors reach 11.3 m and 9.3 m, respectively. RAPF achieves lowest localization error.

**Figure 14:**
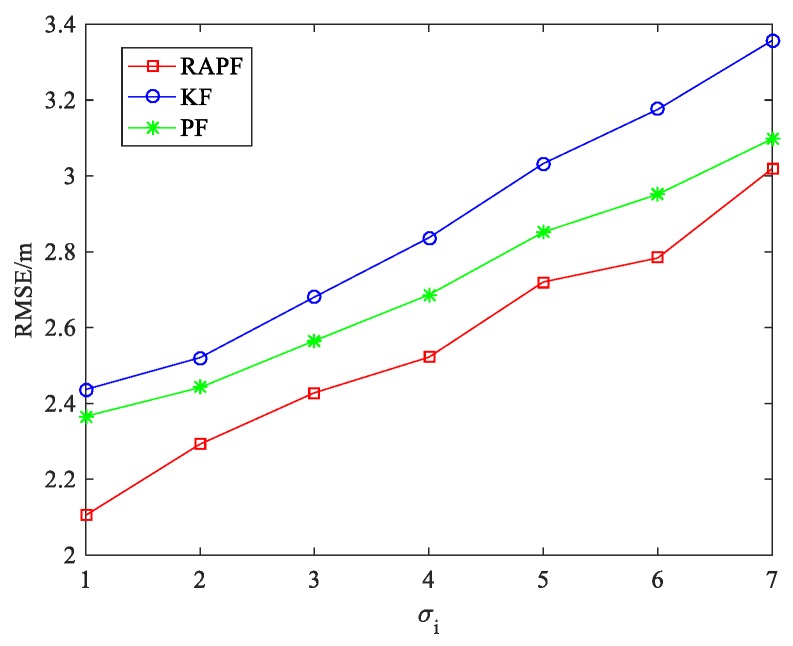
The RMSE versus $\sigma_{i}$.

Figure 14 shows the effect of the measurement noise on localization error. The standard deviation of measurement noise $\sigma_{i}$ increases from 1 to 7. The results demonstrate that performance of the RAPF algorithm is obviously better than that of the other algorithms, but compared with PF, the advantage of the RAPF is not obvious when $\sigma_{i}$ is large. The localization accuracy of RAPF improves 10.824% and 5.749% in comparison with KF and PF.

**Figure 15:**
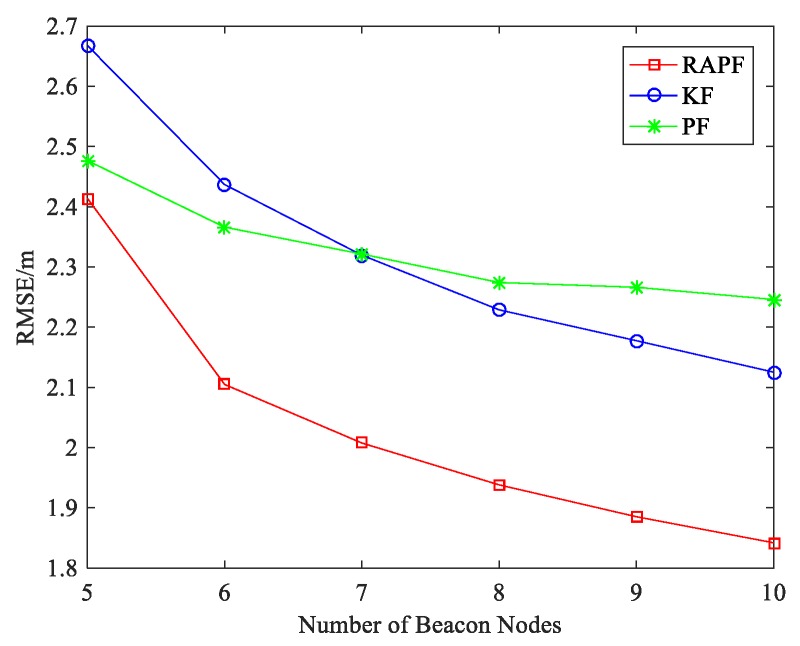
The RMSE versus the number of beacon nodes.

Figure 15 shows the localization error when the number of beacon nodes changes. As we can see, the more beacon nodes deployed, the less localization error is obtained. KF and RAPF are greatly affected by the number of beacon nodes. Among the three algorithms, RAPF always achieves the lowest error. When the number of beacon nodes is 10, the localization accuracy of RAPF improves 12.637% and 12.605%, compared with KF and PF.

**Figure 16:**
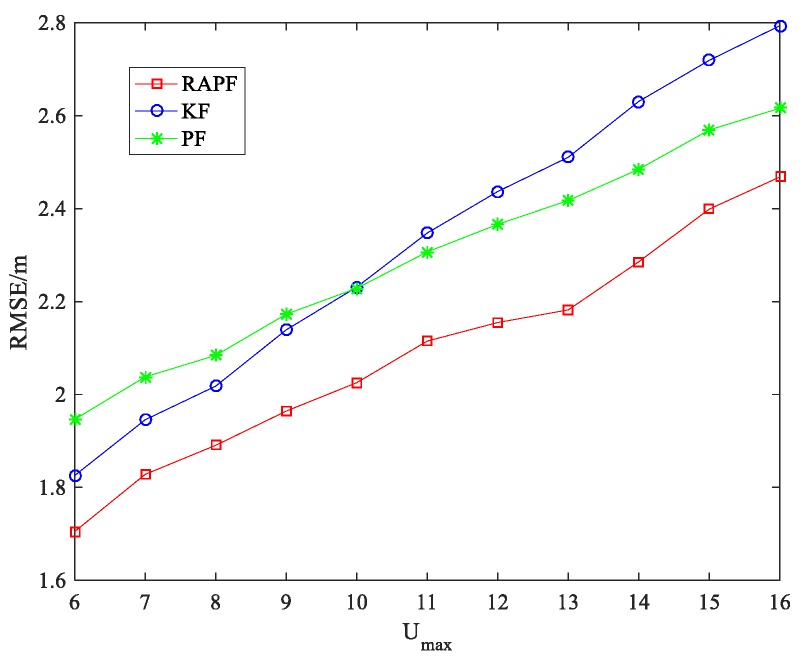
The RMSE the $U_{\max}$.

The parameter of NLOS error is considered in Figure 16. The performance of the three algorithms degrades when the $U_{\max}$ increase. RAPF shows the best localization result. It improves 10.270% and 8.963% in comparison with KF and PF. 

#### 5.1.3. The NLOS Errors Obey Exponential Distribution

The default parameter values in the simulation are shown in Table 4.

**Figure 17:**
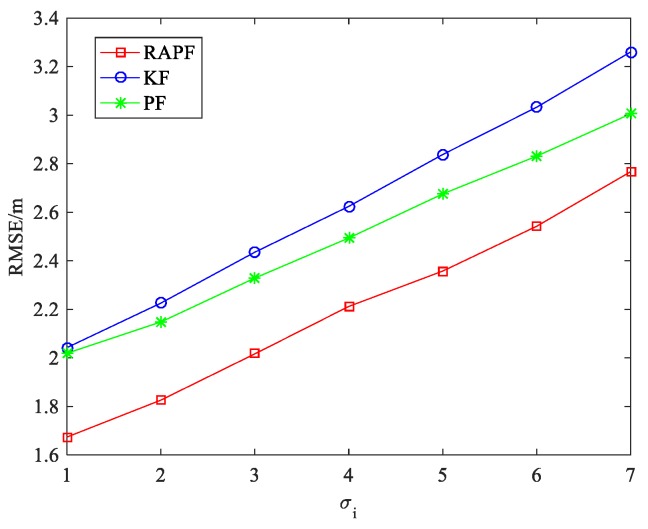
The RMSE versus $\sigma_{i}$.

**Figure 18:**
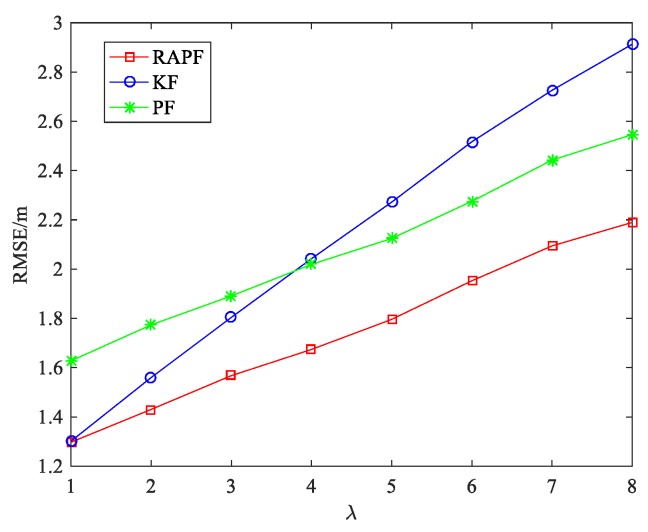
The RMSE versus the parameter λ.

Figure 17 and Figure 18 show the localization results when the NLOS error obeys the experimental distribution, i.e., $\varepsilon_{NLOS}\sim E\left(\lambda \right)$. Figure 17 shows the effect of the standard deviation of the measurement noise $\sigma_{i}$ on the RMSE. It can be observed that the localization performance of KF is similar to that of the PF when $\sigma_{i}$. is small ($\sigma_{i}\;=\;1$). The localization errors of the three algorithms increase when the measurement noise increases. The RAPF algorithm displays the best localization accuracy.

Figure 18 illustrates the impact of parameter λ on the localization performance between the RAPF and other methods. When the NLOS effect is weak (λ=1), the performance of the RAPF is similar to that of the KF. However, the KF algorithm is greatly affected by the parameter λ. The PF and RAPF algorithm are comparatively stable. When the NLOS effect is strong (λ = 8), the performance of the RAPF improves 24.829% and 13.995% compared with KF and PF.

#### 5.1.4. Computational Complexity Analysis

In this subsection, we analyze the computational complexity of the three algorithms. The three algorithms are codeds using Matlab R2016a. The test platfoem is a Windows 10 Professional PC with Intel(R) Core(TM) i7-7700 CPU @ 3.60 GHz and 8.00 GB RAM.

We use the running time to illustrate the computational complexity. The running time is calculated through the MATLAB TIC_TOC functions for elapsed time. TIC_TOC is a directory of MATLAB programs which consider the MATLAB tic and toc functions for computing elapsed time.

As shown in Table 5, the KF algorithm has the lowest computational complexity. The PF algorithm has the highest running time. This is because PF must take some time to generate and adjust particles. Every node in the PF generates its own particles resulting in the highest running time of PF. In RAPF, all beacon nodes use a set of particles together. The computational complexity of RAPF is relatively moderate.

### 5.2. Experiment Results

**Figure 19:**
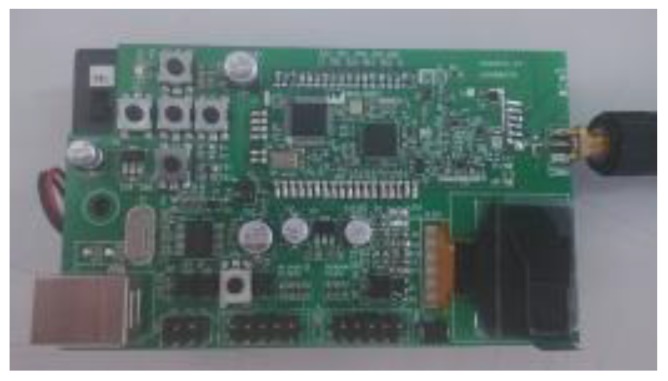
CSS node.

To verify the localization performance of RAPF, we design a real experiment in a practical environment. The chirp spread spectrum (CSS) localization system which is built with a nanoLOC chip is used to obtain the measurements. The CSS node is presented in Figure 19.

**Figure 20:**
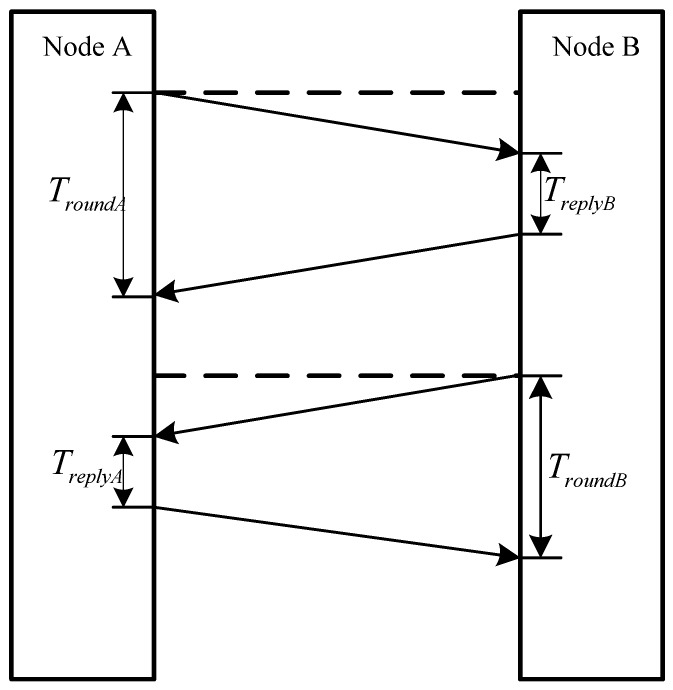
The progress of measuring for CSS localization system.

As shown in Figure 20, the progress of measuring between beacon node and the mobile node in CCS localization system is as follows:

Firstly, node A sends a data package to node B. As the data package is received in node B, it takes some time for node B to deal the data package and reply an acknowledgement to A. The time spent by node B is represented as $T_{replyB}$. When the acknowledgement is received, node A calculates the time which is represented as $T_{roundA}$.

Secondly, node B sends a data package to node A. As the data package is received in node A, it takes some time for node A to deal the data package and reply an acknowledgement to B. The time spent by node A is represented as $T_{replyA}$.

Thirdly, when node B receives the acknowledgement, it calculates the time which is represented as $T_{roundB}$ and sends it to node A.

Finally, based on the four measurements the time of propagation between node A and node B can be given by:

(17) $$T=\frac{\left(T_{roundB}-T_{replyA}\right)+\left(T_{roundA}-T_{replyB}\right)}{4}$$

**Figure 21:**
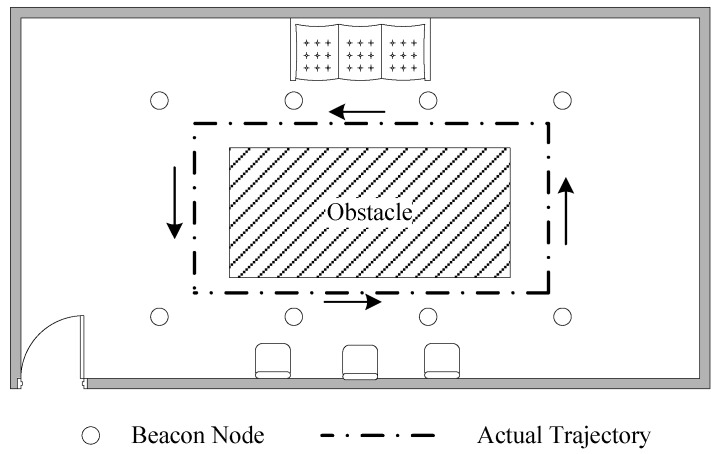
The practical environment.

As shown in Figure 21, there are eight beacon nodes and one unknown node deployed in a room for our experiment. The beacon nodes are deployed around the desk. The desk is the obstacle which causes NLOS propagation. In this experiment, the unknown node moves outside the desk for one loop. Forty sample points are obtained. In this experiment, the measurement frequency of CSS nodes is 20 Hz. The average of the 20 distance measurements can be obtained at each sampling point in order to weaken the adverse impacts imposed to localization accuracy. When the measurements are obtained, this localization algorithm is applied with the measurements using MATLAB to testify the localization performance in practical environments.

**Figure 22:**
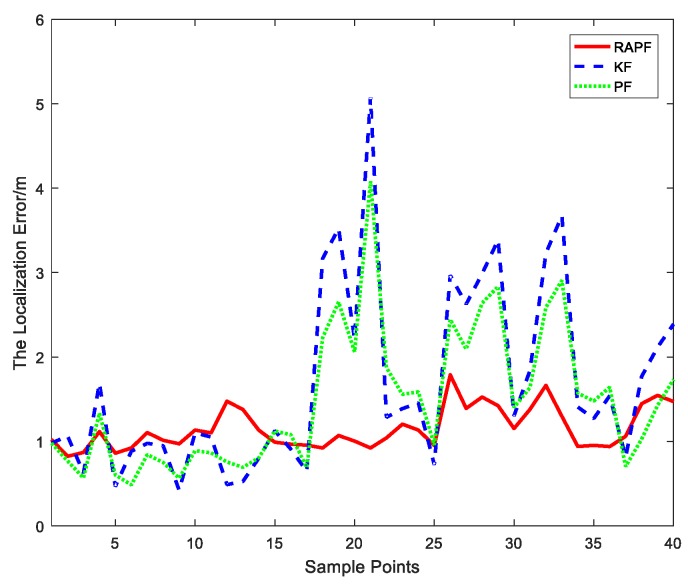
Localization error at each sample points.

**Figure 23:**
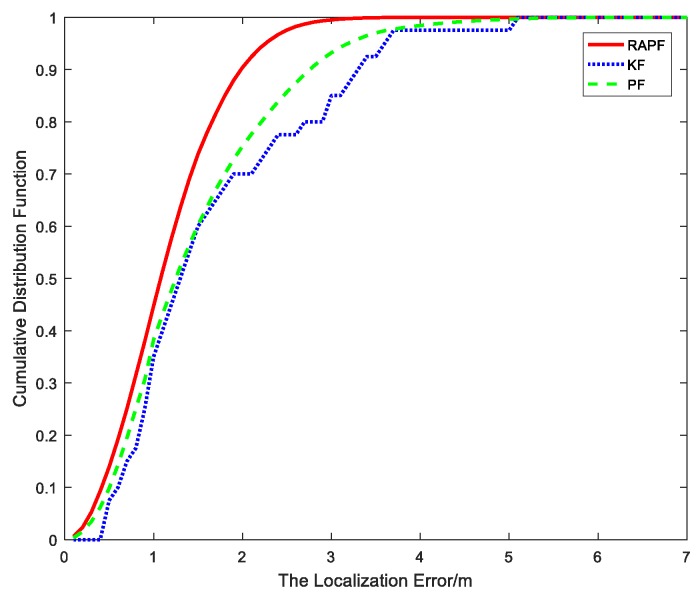
The CDF versus the localization error.

After the measurements are collected, we compare the localization error of the proposed algorithm and the other two algorithms. The localization error and its CDF are represented in Figure 22 and Figure 23. The average localization error of RAPF is 1.1521 m. The localization error of KF and PF reach 1.6718 m and 1.4735 m, respectively. Analyzing the result, we can see RAPF achieves a lower localization error than the other two algorithms. It improves about 31.08% compared to KF and 21.81% compared to PF. As shown in Figure 23, ninety percent of the localization in RAPF is less than 2 m. In contrast, the Kalman filter and particle filter values are 3.2 m and 2.7 m, respectively.

## 6. Conclusions

In this paper, we have proposed a residual analysis-based particle filter algorithm. The proposed algorithm takes the particle filter as a main body. Residual analysis is used in this algorithm to enhance the accuracy of the localization results. RAPF uses residuals to weight particles and select reasonable particles. The residual of particle is fully used in this algorithm. Besides, the proposed algorithm doesn’t make any assumption on the distribution of the NLOS error which means RAPF is robust to any type of NLOS errors. The simulation result and the experiment result confirm the robustness of RAPF. Moreover, RAPF estimates the distance from the unknown node to beacon nodes in this paper, it also can be used to estimate the coordinate of the target. In the future, we will attempt to investigate the performance of the proposed algorithm in RSS and AOA localization. Moreover, the implementation of the proposed method in a distributed way is another inteersting further research topic.

## Figures and Tables

**Table 1:** List of key notations.

Notation	Explanation	Notation	Explanation
N	the number of beacon nodes	$\left[x_{i},y_{i}\right]$	the coordinates of beacon nodes
[x,y]	the position of unknown node	ε	the measurement error
${\hat{d}}_{i}^{k}$	the measured distance measurement of the i-th beacon node at time *k*	$d_{i}^{k}$	the true distance between the i-th beacon node and the unknown node at time *k*
$\varepsilon_{NLOS}$	the NLOS error	$\sigma_{i}^{2}$	the deviation of measurement error
P	the number of particles	$\left[X_{p}^{k},Y_{p}^{k}\right]$	the coordinate of particles at time *k*
$d_{i,p}^{k}$	the true distance between the i-th beacon node and the p-th particle at time *k*	$W_{i,p}^{k}$	the weight of p−th particle to i−th beacon node at time *k*
$res_{i,p}^{k}$	the residual of the p−th particle to the i−th beacon node at time *k*	$res_{p}^{k}$	the sum of the residuals of the p−th particle to all the beacon node at time *k*
S	the number of the reserved particles after the first selection	Q	the number of the reserved particles after the second selection
λ	the estimated NOLS error probability	L	the number of the reserved particles after two-time selection
$NW_{p}^{k}$	the weight of p−th particle to i−th beacon node at time *k* after normalization	$dis_{i}^{k}$	the estimated distance from the unknown node to the i−th beacon node at time *k*
$\sigma_{p}^{2}$	the deviation of particle movement		

**Table 2:** The default parameter values.

Parameters	Symbol	Default Values
The number of beacon nodes	N	6
The probability of LOS propagation	α	0.6
The standard deviation of measurement noise	$\sigma_{i}$	1
The NLOS error	$N\left(\mu_{NLOS},\sigma_{NLOS}^{2}\right)$	$N\left(4,6^{2}\right)$
The standard deviation of particle movement	$\sigma_{p}^{2}$	3
The number of sample points	T	100
The number of Monte Carlo runs	$t_{n}$	1000

**Table 3:** The default parameter values.

Parameters	Symbol	Default Values
The number of beacon nodes	N	6
The probability of LOS propagation	α	0.6
The standard deviation of measurement noise	$\sigma_{i}$	1
The NLOS error	$U\left(U_{\min},U_{\max}\right)$	U(2,12)
The standard deviation of particle movement	$\sigma_{p}^{2}$	3
The number of sample points	T	100
The number of Monte Carlo runs	$t_{n}$	1000

**Table 4:** The default parameter values.

Parameters	Symbol	Default Values
The number of beacon nodes	N	6
The probability of LOS propagation	α	0.6
The standard deviation of measurement noise	$\sigma_{i}$	1
The NLOS error	E(λ)	E(4)
The standard deviation of particle movement	$\sigma_{p}^{2}$	3
The number of sample points	T	100
The number of Monte Carlo runs	$t_{n}$	1000

**Table 5:** The running times.

Algorithms	Running Times
KF	0.01196 s
PF	0.12832 s
RAPF	0.05548 s
